# Information Theory for Human and Social Processes

**DOI:** 10.3390/e23010009

**Published:** 2020-12-23

**Authors:** Martin Hilbert

**Affiliations:** Department of Communication, G.G. Computer Science, University of California, 370 Kerr Hall, 1 Shields Avenue, Davis, CA 95616, USA; hilbert@ucdavis.edu

Ever since its earliest years, information theory has enjoyed both a promising and complicated relationship with the social sciences. Shannon himself applied his “mathematical theory of communication” to human communication early on, allegedly having his wife Betty estimating word probabilities to calculate the first approximation of the entropy of English [1]. Five years later, he then warned of a “scientific bandwagon”, saying that information theory “is not necessarily relevant to such fields as psychology, economics, and other social sciences”. He added that he personally still believed “that many of the concepts of information theory will prove useful in these other fields—and, indeed, some results are already quite promising—but the establishing of such applications is not a trivial matter of translating words to a new domain, but rather the slow, tedious process of hypothesis and experimental verification” [2].

It turned out that in the social sciences, this translation process was indeed slow and tedious. After Miller’s famous “magical number seven” paper [3] and Attneave’s groundbreaking “Applications of Information Theory to Psychology” [4], the emerging field of Communication adopted information theoretic concepts to study group decision-making [5,6,7,8], relational control in relationships [9,10,11], mass communication [12], and talk and silence sequences in conversations [13,14,15]. A main historical account for why this work was discontinued was that “gathering everyday conversations … is nearly impossible … unless one carries a tape recorder around all day (a cumbersome and hardly practical endeavor)” [10]. Additional culprits are the “adoption of approaches from other fields such as psychology that do not emphasize process as much as communication” [16], “the perceived scope of effort required from the researcher” [17], and those dynamics were “simply impractical to compute” [4] before today’s computing power.

While these rather practical and computational limitations have been overcome in recent years due to the “big data” flood and omnipresent cloud computing, unfortunately, there have also been conceptual reservations to the applicability of information theory to the social sciences, especially during the 1990s and early 2000s. It was argued that information theory was supposedly “inappropriate to represent social processes” [18] as it was allegedly a “misleading misrepresentation of the nature of human communication” [19]. It is striking that all of these critics refer to Shannon’s channel logic of communication as the “Shannon–Weaver model” [20]. In 1949, in his role as science advocate, Warren Weaver asked Shannon to reprint his two-part paper from 1948 [21] in book format. He added a 28-page introduction for the 144-page book and changed the title from “A Mathematical Theory…” to “The Mathematical Theory…” [22]. Weaver sees his introduction as “an interpretation of mathematical papers by Dr. Claude E. Shannon” [22] and not as an original contribution. Given this consistent misattribution of credits, it is questionable how familiar these critical social scientists indeed were with Shannon’s comprehensive framework and what became of it during the subsequent decades [23,24]

Reaching the year 2020, the increase in human interactions taking place in digital environments has led to a refound fascination with applying information theory in the social sciences. The new abundance of behavioral “big data” and our computational resources allow researchers to even calculate measures that converge rather slowly, while, at the same time, the maturation of the social sciences has led to an increased interest in more sophisticated nonlinear methods and measures.

This Special Issue compiles 11 creative research articles on innovative uses of information theory and its extensions to better understand human behavior and social processes. The articles in this Special Issue are proof of the abundant opportunities offered by information theory to better understand the nature of humans and its societal systems and dynamics.

Hilbert and Darmon [25] use information theory to explain “How Complexity and Uncertainty Grew with Algorithmic Trading”. On the micro level, traders employ algorithms to reduce their local uncertainty by creating more complex algorithmic patterns. This entails a more predictable structure and more complexity. On the macro level, the increased overall complexity implies more combinatorial possibilities and therefore more uncertainty about the future. They use information theory’s expansion known as computational mechanics to resolve the seeming contradiction between decreased uncertainty on the micro level and the simultaneously increased uncertainty on the macro level.

Uppal, Ferdinand, and Marzen [26] are “Inferring an Observer’s Prediction Strategy in Sequence Learning Experiments”, which link to Shannon’s own historic quest to understand human prediction capabilities [1]. The predictive brain is the dominant framework in cognitive science today, viewing humans or other animals as prediction machines. The authors offer a way to uncover what is under the hood of the human prediction machine. They show that with only one observer, one can infer the model class used by the human predictor (generalized linear model vs. Bayesian model) but not necessarily the model’s parameters.

Two studies in this Special Issue use MaxEnt and spatial information entropy methods to explore the spatial–temporal evolutionary characteristics of ethnic groups to better understand the different stages of their transition and migration, including “Spatial–Temporal Characteristic Analysis of Ethnic Toponyms Based on Spatial Information Entropy at the Rural Level in Northeast China” [27] and “Using the Maximal Entropy Modeling Approach to Analyze the Evolution of Sedentary Agricultural Societies in Northeast China” [28]. Related in its geographical theme, but of less historic and more current societal interest, Lenormand and colleagues [29] use a mobile phone dataset and an entropy-based metric to measure the attractiveness of a location in the Rio de Janeiro Metropolitan Area (Brazil) as the diversity of visitors’ location of residence (“Entropy as a Measure of Attractiveness and Socioeconomic Complexity in Rio de Janeiro Metropolitan Area”).

In “Economics of Disagreement—Financial Intuition for the Rényi Divergence”, Andrei Soklakov [30] shows how a large class of models for human disagreements can be transformed into prototypical models of investment opportunities. This offers a market-like social mechanism to resolve disagreements, whereas funds flow naturally through optimized investments to support a more accurate view.

In the “Source of Knowledge Dynamics—Transition from High School to University”, Hadad and colleagues [31] make innovative use of Markov chains to model the ongoing dynamics in the educational transition from printed books and libraries to online materials.

“An Objective-Based Entropy Approach for Interpretable Decision Tree Models in Support of Human Resource Management: The Case of Absenteeism at Work” [32] uses information theory to uncover subgroups of employees with common characteristics and a similar level of absenteeism.

Wiener [33] uses the maximum entropy principle to make inferences about the unobserved mobility decisions of workers in U.S. household data in “Labor Market Segmentation and Immigrant Competition: A Quantal Response Statistical Equilibrium Analysis”. His model captures a substantial proportion of the informational content of observed wage distributions.

Of course, Integrated Information Theory’s (IIT) ambition to mathematically model consciousness cannot be missed in a current discussion of information theory for human processes. Popiel and colleagues use the generalized Ising model to calculate Φ as a function of temperature in toy models of fully connected neural networks. In “The Emergence of Integrated Information, Complexity, and ‘Consciousness’ at Criticality” [34], they show that IIT’s Φ can capture integrated information in an empirical dataset, which is promising for future applications of IIT to empirical social science data.

The biggest methodological contribution to the social sciences in this Special Issue comes from Dave Darmon: “Discrete Information Dynamics with Confidence via the Computational Mechanics Bootstrap: Confidence Sets and Significance Tests for Information-Dynamic Measures” [35]. It solves an important issue in the practical application of an expansion of information theory referred to as “computational mechanics” [36,37,38]. Computational mechanics derives optimized minimal sufficient statistics of temporal empirical data in the form of hidden Markov models known as ε-machines. In short, it is a “white-box” machine learning technique that allows social theorists to model societal dynamics [39,40,41]. As with any empirical inference tool, it is important to obtain an idea of confidence sets for hypothesis tests of the underlying measures. The only existing method, until now, was a Bayesian inference method, which is computationally quite demanding [42]. Darmon provides a bootstrap method for constructing confidence sets and significance tests for the popular Causal State Splitting Reconstruction (CSSR) algorithm to derive ε-machines [43]. Given the omnipresent importance of significance tests in the social sciences, this contribution opens new doors for the future application of information theory to social science research.

In closing, in line with Darmon’s contribution, it is important to state that there are still many outstanding methodological challenges for the application of information theory to the social sciences. Maybe the most important one is the advancement of multivariate information theory. Social systems are inherently interconnected systems consisting of many interrelated parts. Therefore, still, the predominant preference for empirical methods in the social sciences are those that allow working with a considerable number of variables, including multiple regression, ANCOVA and MANCOVA, and structural equations models. It is not uncommon that a single model includes more than a dozen variables. In information theory, the main workhorse is still Shannon’s bivariate setup of the sender and receiver, while a third variable still creates much confusion among scholars. The leading textbook laments that “unfortunately, [the three variable mutual information] is not necessarily nonnegative” [23], while the second most common textbook recommends against illustrating entropy of three or more variables in the graphical form [24]. Important advancements in multivariate information theory and information decomposition was showcased, among other outlets, by another recent Special Issue in *Entropy* [44]. These advancements in the expansion and solidification of the mathematical aspects of information theory provide future promises for even more applications of information theory for human and social processes.
